# Structural insights into the species preference of the influenza B virus NS1 protein in ISG15 binding

**DOI:** 10.1007/s13238-018-0598-4

**Published:** 2018-12-05

**Authors:** Yinan Jiang, Xinquan Wang

**Affiliations:** The Ministry of Education Key Laboratory of Protein Science, School of Life Sciences, Beijing Advanced Innovation Center for Structural Biology, Collaborative Innovation Center for Biotherapy, Tsinghua University, Beijing, 100084 China

**Dear Editor,**

Type I interferons (IFNs) are secreted in the context of viral infection and mediate the expression of more than 300 IFN-stimulated genes (Schoggins and Rice, 2011). One of the earliest and most highly induced ISGs is IFN-stimulated gene 15 (ISG15), a ubiquitin-like protein (Ubl) comprising two Ubl domains connected by a linker (Farrell et al., 1979; Haas et al., 1987; Narasimhan et al., 2005). Similar to ubiquitin, ISG15 can be covalently attached to lysine on numerous target proteins via its C-terminal LRLRGG sequence in a process called ISGylation, which is catalyzed by the E1-activating enzyme Ube1L, the E2 conjugating enzyme UbcH8 and E3 ligases such as human HerC5 and mouse HerC6 (Hermann and Bogunovic, 2016).

ISG15 was shown to exhibit potent antiviral activity against influenza B virus in mouse model (Lenschow et al., 2007). Both ISG15^−/−^ and Ube1L^−/−^ mice were more susceptible to infection with influenza B virus, establishing that ISGylation inhibits influenza B virus replication (Lai et al., 2009). Interestingly, it was reported that NS1B exhibits human-specific binding to ISG15 and potently antagonizes human but not mouse ISGylation, which provides one explanation for the restriction of influenza B virus to humans (Sridharan et al., 2010; Versteeg et al., 2010). However, the molecular basis for the species-specific binding is not completely understood yet, especially considering the relatively high sequence identities (~50%) among different ISG15 proteins.

To investigate whether NS1B could interact with ISG15 proteins from other mammalian species, we conducted gel filtration analysis of NS1B-NTR, containing the 103 N-terminal residues of NS1B, and ISG15 proteins from different mammalian species. As expected, the elution peak of human ISG15 (hISG15) and NS1B-NTR mixture was shifted toward the higher molecular weight end of the profile, indicating the formation of the complex (Fig. 1A). Unexpectedly, NS1B-NTR was also observed to migrate to a higher molecular weight when mixed with murine ISG15 (mISG15), canine ISG15 (cISG15) and bovine ISG15 (bISG15), suggesting that NS1B-NTR could form stable complexes with ISG15 proteins from other mammalian species (Fig. 1B–D).

**Figure 1:**
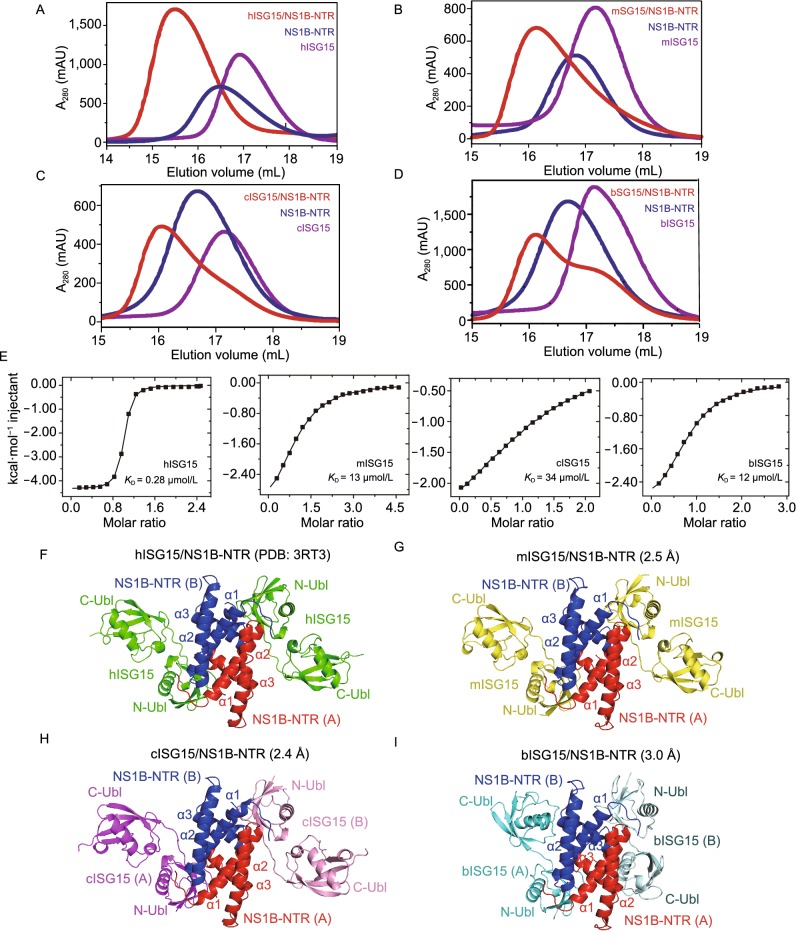
**Overall structures of different ISG15/NS1B-NTR complexes**. (A–D) Size exclusion profiles showing the complex formation upon mixing NS1B-NTR with hISG15 (A), mISG15 (B), cISG15 (C) or bISG15 (D). (E) Measurement of the binding affinities between NS1B-NTR and different ISG15 proteins by ITC (from left to right). (F) Previously determined hISG15/NS1B-NTR complex structure (PDB code: 3RT3), in which the asymmetric unit contains one NS1B-NTR monomer and one hISG15 molecule. (G–I) Structures of the mISG15/NS1B-NTR (G), cISG15/NS1B-NTR (H) and bISG15 /NS1B-NTR (I) complexes determined in this study. The crystallographic asymmetric unit of mISG15/NS1B-NTR contains one NS1B-NTR monomer and one mISG15 molecule. The cISG15/NS1B-NTR and bISG15/NS1B-NTR crystallographic asymmetric units contain one NS1B-NTR dimer with two cISG15 and bISG15 molecules, respectively. Red, NS1B-NTR monomer A; blue, NS1B-NTR monomer B; green, hISG15; yellow, mISG15; magenta, cISG15 molecule A; cyan, cISG15 molecule B; cyan, bISG15 molecule A; pale cyan, bISG15 molecule B

We next performed isothermal titration calorimetry (ITC) studies to measure the binding affinities of NS1B-NTR for different ISG15 proteins. NS1B-NTR exhibited a dissociation constant (*K*_D_) of 0.28 μmol/L with hISG15, while the binding affinities of NS1B-NTR for mISG15, cISG15 and bISG15 were 13, 34 and 12 μmol/L, respectively (Fig. 1E). The binding stoichiometry between NS1B-NTR and different ISG15 proteins was 1:1 determined by ITC (Table S1), and it has been reported that NS1B-NTR exists as dimer in solution, thus it can be inferred that NS1B-NTR dimer binds two copies of ISG15 molecules in solution (Guan et al., 2011).

The crystal structure of hISG15/NS1B-NTR complex has been determined by our and other groups (Fig. 1F) (Guan et al., 2011; Li et al., 2011). To elucidate the molecular basis for the binding of other ISG15 proteins by NS1B-NTR and the differences in their binding affinities, we solved the crystal structures of NS1B-NTR in complex with mISG15, cISG15 and bISG15 at resolutions of 2.5 Å, 2.4 Å and 3.0 Å, respectively (Table S2). These four complexes exhibited a very similar architecture, in which one closely packed NS1B-NTR dimer utilizes two symmetric sites to bind two ISG15 molecules (Fig. 1G–I). Compared to the NS1B-NTR dimer in the free form, there was a significant conformational change in the C-terminal tail of NS1B-NTR monomer as a result of its binding to the ISG15 protein (Fig. S1A and S1B).

As two separate rigid bodies, the N-Ubl and C-Ubl domains are structurally conserved among different ISG15 proteins (Fig. S1C and S1D). However, structural comparisons of the full-length ISG15 structures revealed an intrinsic flexibility between these two Ubl domains. The alignment of the two cISG15 molecules and the two bISG15 molecules in one asymmetric unit based on N-Ubl showed an approximately 5-degree rotation and 14-degree rotation between C-Ubl domains, respectively (Fig. S2A and S2B). When we compared the structures of different ISG15 proteins, bISG15 was found to be exceptional in the positional change of the C-Ubl (Fig. S2C). Notably, the bISG15 linker connecting the two Ubl domains is the shortest with only three residues, whereas other ISG15 proteins all have a six-residue linker (Fig. S3).

Similar to the previously observed interface between NS1B-NTR and hISG15, there are two binding sites, designated I and II, between NS1B-NTR and either mISG15, cISG15 or bISG15 (Fig. S4A–D). In the binding site I, a protruding loop between the β1 and β2 strands in the N-Ubl of ISG15 (residues M9–G12 in hISG15, residues M9–G12 in mISG15, residues M15–G18 in cISG15 and residues M9–G12 in bISG15) inserts into a groove between two NS1B-NTR monomers (Fig. 2A). Residues involved in the interactions with a distance cutoff of 4.0 Å are listed in Tables S3–5. The major chemical feature of the binding site I is a hydrophobic core consisting of a leucine residue (Leu10 in hISG15, mISG15 and bISG15; Leu16 in cISG15) from ISG15, in conjunction with Met84, Val87 and Leu88 from NS1B-NTR (Fig. 2A). This leucine residue is conserved in different ISG15 proteins, and mutating it to alanine in hISG15 caused a dramatic ~60-fold drop of affinity for NS1B-NTR, from 0.28 μmol/L to 17 μmol/L (Figs. 2B and S3). Moreover, none of the leucine to alanine mutants of mISG15, cISG15 and bISG15 showed detectable interactions with NS1B-NTR (Fig. 2B).

**Figure 2:**
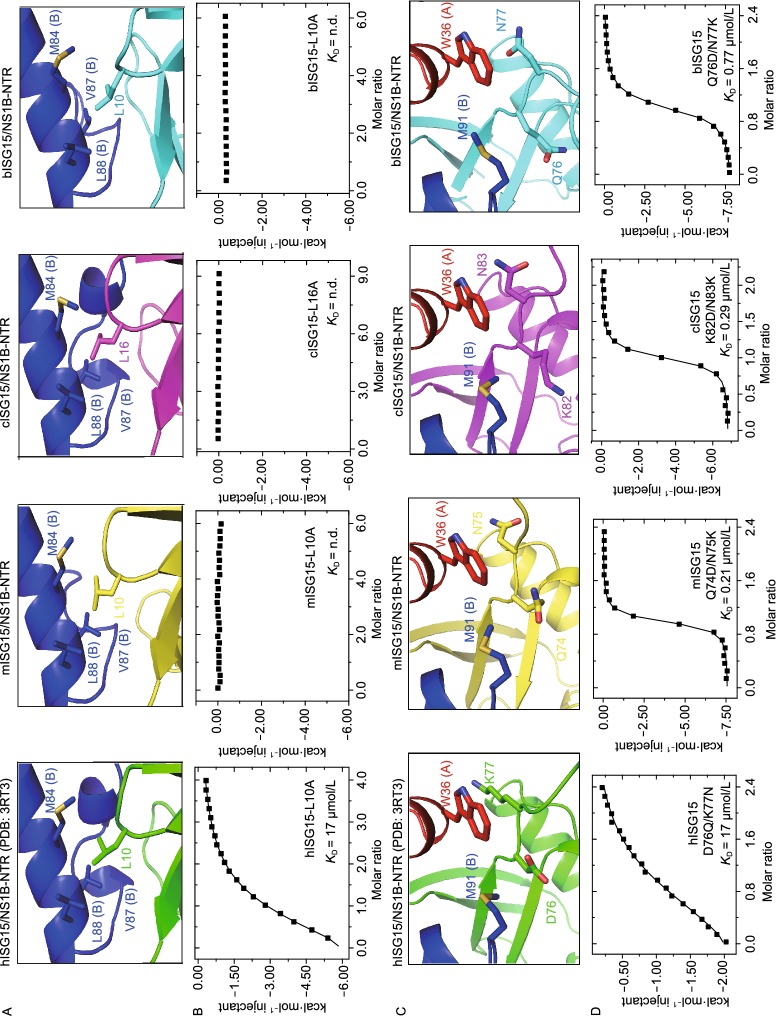

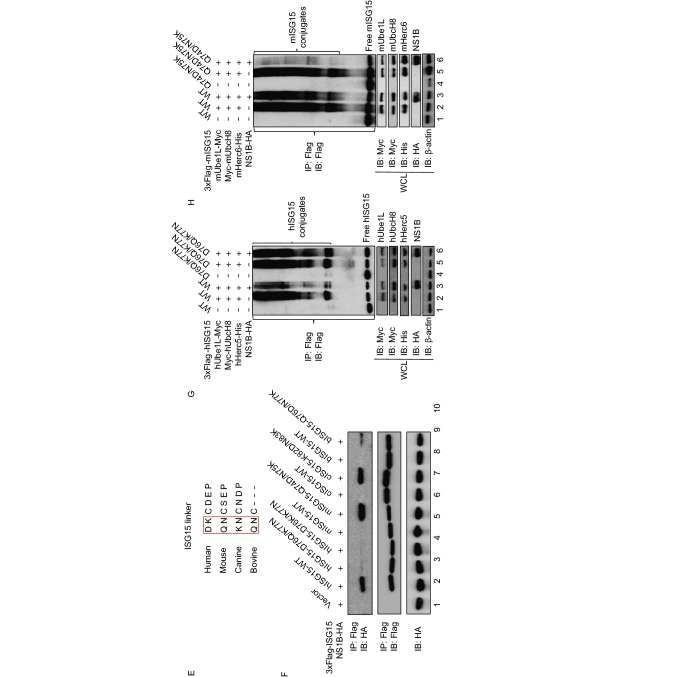
**The binding interfaces in ISG15/NS1B-NTR complexes**. (A) The conserved leucine at binding site I in the N-Ubl domain of ISG15 and the surrounding interacting residues of NS1B-NTR in the pocket of hISG15/NS1B-NTR (PDB: 3Rt3), mSIG15 /NS1B-NTR, cISG15/NS1B-NTR, and bISG15/NS1B-NTR crystal structures (from left to right). (B) Measurement of the binding affinities between NS1B-NTR and hISG15-L10A, mISG15-L10A, cISG15-L16A, bISG15-L10A by ITC (from left to right). (C) The first two residues in the linker of ISG15 have van der Waals contacts with Met 91 and Trp 36 of the NS1B-NTR in hISG15/NS1B-NTR (PDB: 3Rt3), mSIG15/NS1B-NTR, cISG15/NS1B-NTR, and bISG15/NS1B-NTR crystal structures (from left to right). (D) Measurement of the binding affinities between NS1B-NTR and hISG15-D76Q/K77N, mISG15-Q74D/N75K, cISG15-K82D/N83K, and bISG15-Q76D/N77K by ITC (from left to right). (E) Sequence alignment of ISG15 linker from the indicated mammalian species. The first two residues in ISG15 linker were shown in bold and boxed in red. (F) NS1B-HA and 3xFlag-ISG15 were co-expressed in HEK293T cells and the immunoprecipitates were analyzed by immunoblots probed with anti-Flag and anti-HA antibodies. (G) Human E1, E2, E3 and 3xFlag-ISG15 plasmids were co-transfected in HEK293T cells with plasmid encoding NS1B-HA. The immunoprecipitates were analyzed by Western blot using anti-Flag, anti-HA, anti-Myc and anti-His antibodies. Detection of β-actin was used as a loading control. (H) Mouse E1, E2, E3 and 3xFlag-ISG15 plasmids were co-transfected with plasmid encoding NS1B-HA. The immunoprecipitates were analyzed by immumoblots as described for panel (G). Red, NS1B-NTR monomer A; Blue, NS1B-NTR monomer B; green, hISG15; yellow, mISG15; magenta, cISG15; cyan, bISG15

The binding site II at hISG15/NS1B-NTR interface is located around the first two residues in hISG15 linker (Fig. S4A). Although the linker exhibits flexibility in different ISG15 proteins, their interaction patterns with NS1B-NTR at site II are very similar. At the interface, the first linker residue hISG15 Asp76, mISG15 Gln74, cISG15 Lys82 and bISG15 Gln76 all have van der Waals contacts with Met91 of NS1B-NTR (Fig. 2C). The second residue in the linker region is asparagine in mISG15 (Asn75), cISG15 (Asn83) and bISG15 (Asn77), which interacts with Trp36 in NS1B-NTR (Fig. 2C). This residue is replaced with Lys in hISG15, which forms a cation-π interaction with Trp36 in NS1B-NTR. Because the binding site I is highly conserved, it is tempting to suggest that the interactions around the first two residues in ISG15 linker are responsible for the stronger binding between NS1B-NTR and hISG15. To confirm this hypothesis, we converted the first two residues in each of the ISG15 proteins to alanine. The mutated hISG15 showed a dramatic decrease of the binding affinity for NS1B-NTR, from 0.28 μmol/L to 5 μmol/L (Fig. S5A). By contrast, the same mutations did not significantly affect the binding between NS1B-NTR and mISG15, cISG15 or bISG15 (Fig. S5B–D). Remarkably, when we substituted the Asp76 and Lys77 in hISG15 with mouse residues Gln and Asn, respectively, the binding was dramatically decreased to 17 μmol/L, comparable with the 13 μmol/L binding affinity between NS1B-NTR and mISG15 (Fig. 2D). More interestingly, when we mutated these two residues in mISG15, cISG15 and bISG15 to their respective counterparts (Asp76 and Lys77) in hISG15, the binding affinities increased to 0.21, 0.29 and 0.77 μmol/L, respectively (Fig. 2D).

Next, we performed co-immunoprecipitation (Co-IP) and ISGylation assays in HEK293T cells co-transfected with full-length NS1B and wild-type ISG15 proteins or ISG15 mutants bearing mutations at the first two residues in the linker (Fig. 2E) The Co-IP experiment showed that NS1B exhibited much stronger binding to hISG15 than mISG15, cISG15 and bISG15 (Fig. 2F, lanes 2, 5, 7 and 9). Substituting the first two residues in hISG15 linker with their counterparts in mISG5/bISG15 or cISG15 decreased NS1B binding to a much weaker level that was nearly undetectable in the western blot (Fig. 2F, lanes 3 and 4). Remarkably, mISG15-Q74D/N75K, cISG15-K82D/N83K and bISG15-Q76D/N77K acquired strong NS1B binding by replacing the two residues in the linker at site II with their hISG15 counterparts (Fig. 2F, lanes 6, 8 and 10). In the ISGylation assay, the expression of NS1B potently reduced hISG15 conjugation and only slightly decreased mISG15 conjugation (Fig. 2G, lanes 1–3, Fig. 2H, lanes 1–3). However, by replacing the two residues in hISG15 linker at site II with their mouse counterparts, NS1B only had minimal effects on hISG15-D76Q/K77N conjugation (Fig. 2G, lanes 4–6). In contrast, substituting the two residues in mISG15 linker at site II with their hISG15 counterpart resulted in strikingly less mISG15-Q74D/N75K conjugation (Fig. 2H, lanes 4–6).

The previous conclusion of species-specific binding of NS1B to ISG15 was based on GST pull-down of the cell lysates of transfected HEK293T cells, showing that only GST-hISG15 was able to pull down NS1B (Sridharan et al., 2010; Versteeg et al., 2010). Actually, we also only observed co-immunoprecipitation of NS1B with hISG15 in our Co-IP assay. The weak binding between NS1B and non-human ISG15 proteins may account for the failure of detecting the interactions in the transfected cells. However, currently we cannot exclude the possibility that other cellular factors may affect the NS1B/ISG15 interaction, which needs to be clarified in the future study.

Although our current study more accurately revised the nature of the NS1B/ISG15 binding as displaying a manner of species preference, its implications for influenza B host restriction remain elusive. Furthermore, the latest studies indicated that the antiviral activity of ISGylation and the inhibition of ISGylation by NS1B are much more complicated than expected. A recent study has demonstrated that ISGylation is not suppressed by NS1B in influenza B virus-infected human cells. Instead, NS1B binds and sequesters ISGylated viral proteins, primarily ISGylated viral nucleoprotein (NP), which acts as a dominant inhibitor of oligomerization of the more abundant unconjugated counterparts (Zhao et al., 2016). As a result, the inhibition of the formation of viral ribonucleoproteins that catalyze viral RNA synthesis is limited, and hence viral protein synthesis and virus replication are increased. Thus, NS1B may not be able to sequester ISGylated NP efficiently in mice due to the low binding affinity between mISG15 and NS1B, and therefore cannot protect influenza B virus from antiviral response in mice. In stark contrast to conclusions drawn from *in vivo* studies in mice, humans with ISG15 deficiency do not display enhanced susceptibility to viral infection (Bogunovic et al., 2012; Speer et al., 2016). The role that free intracellular ISG15 plays in the context of virus infection could be proviral by binding USP18, which is a potent negative regulator of IFN-α/β signaling, and preventing it from SKP2-mediated degradation (Zhang et al., 2015). In this scenario, NS1B could suppress IFN-α/β signaling by sequestering free ISG15 and increasing the pool of unconjugated ISG15, thereby enhancing USP18 levels to downregulate IFN-α/β signaling. We have found that NS1B-NTR can interact with hISG15/hUSP18 complex and form a tertiary complex *in vitro*, supporting this hypothesis (unpublished data). However, the implication for the formation of the tertiary complex needs to be further investigated.

## Electronic supplementary material

Below is the link to the electronic supplementary material.

Supplementary material 1 (PDF 1316 kb)
