# Supplementary material for: Structural insights into the species preference of the influenza B virus NS1 protein in ISG15 binding
Source: Protein Cell. 2018 Dec 5;10(9):681–7. doi: 10.1007/s13238-018-0598-4 (PMC6711944; doi:10.1007/s13238-018-0598-4)
Supplement: Supplementary file 1 — Supplementary material 1 (PDF 1316 kb) [file 13238_2018_598_MOESM1_ESM.pdf]

SUPPLEMENTARY MATERIALS

Structural insights into the species preference of the influenza B virus NS1 protein in ISG15 binding

Yinan Jiang[1], Xinquan Wang[1,*]

[1]The Ministry of Education Key Laboratory of Protein Science, School of Life Sciences, Beijing Advanced Innovation Center for Structural Biology, Collaborative Innovation Center for Biotherapy, Tsinghua University, Beijing 100084, China

*Correspondence: xinquanwang@mail.tsinghua.edu.cn

To whom correspondence should be addressed: Xinquan Wang, Medical Science Building C226, Tsinghua University, Beijing 100084, P.R.China, Tel: (+) 86-10-62789401, E-mail: xinquanwang@mail.tsinghua.edu.cn

Keywords: ISG15, NS1B, X-ray crystallography, site-directed mutagenesis, binding affinity

Running title:

Crystal structures of different ISG15/NS1B-NTR complexes

MATERIALS AND METHODS

Protein expression and purification

All constructs were generated by using a standard PCR-based cloning strategy. To address the disulfide-dependent instability of the ISG15 proteins, the conserved cysteine in the linker region of all ISG15 molecules was mutated to serine, yielding human ISG15-C78S, murine ISG15-C76S, canine ISG15-C84S, and bovine ISG15-C78S. A recombinant full-length ISG15 with a C-terminal LRLRGG sequence

and NS1B-NTR (1-103) were cloned into the pGEX-6p-1 vector (GE Healthcare, USA) with a glutathione S-transferase (GST) tag at the N terminus. The plasmids were then introduced into *Escherichia coli* strain BL21 (DE3) (Thermo Fisher Scientific, USA) and overexpressed by induction with 0.5 mM isopropyl-β-D-thiogalactopyranoside (IPTG) for 16 h at 16 ℃. The bacteria were then pelleted by centrifugation at 4 000 × g for 15 min, resuspended in 10 mM HEPES pH 7.2 with 500 mM NaCl and lysed by sonication. After centrifugation, the supernatant was loaded onto a glutathione-Sepharose 4 Fast Flow column (GE Healthcare, USA). The GST tag was removed using PreScission Protease (GE Healthcare, USA) at 4 ℃ overnight. The protein was further purified by size-exclusion chromatography on a Superdex 200 HR 10/30 column (GE Healthcare, USA). The purified ISG15 and NS1B-NTR were incubated on ice for 30 min and the stable ISG15/NS1B-NTR complex was further polished using a Superdex 200 HR 10/30 size exclusion column (GE Healthcare, USA). All the ISG15 variants were expressed and purified using the same protocol.

Size Exclusion Chromatography

The purified NS1B-NTR was mixed with ISG15 protein from different species at a molar ratio of about 1:1 at 4 ℃ for 30 min, after which the mixture was subjected to size exclusion (Superdex 200, GE Healthcare, USA) for chromatographic analysis in a buffer containing 10 mM HEPES-sodium, 500mM NaCl, pH 7.2. After injection, the samples were eluted at a flow rate of 0.5 mL/min. Samples from relevant fractions

were analyzed via SDS-PAGE and visualized by staining with Coomassie brilliant blue G-250 (Thermo Fisher Scientific, USA).

Crystallization

Protein samples were concentrated to ~15 mg/ml and crystallization was performed in hanging-drop vapor-diffusion experiments at 18 ℃. Crystals were grown by mixing 1μl of the protein sample with 1μl of reservoir solution. mISG15 in complex with NS1B-NTR in the buffer containing 10 mM HEPES, 500mM NaCl, pH 7.2 was used for crystallization. Crystals were obtained with a crystallization solution comprising 0.1 M magnesium chloride, 0.1 M Na-HEPES, pH 7.0, 13 % w/v PEG 4000. For the cISG15/NS1B-NTR complex in the buffer comprising 10 mM HEPES, 150 mM NaCl, pH 7.2, crystals were grown in 0.1 M magnesium chloride, 0.1 M MES, pH 6.7, 16 % w/v PEG 6000. Crystals of the bISG15/NS1B-NTR complex in the buffer containing 20 mM Tris, 100 mM NaCl, pH 8.0 were obtained in 0.1 M MES, pH 6.7 11 % w/v PEG 6000, 5 % v/v MPD. Crystals were cryoprotected in reservoir solution plus 20% (v/v) glycerol and flash-frozen in liquid nitrogen before data collection.

Data collection and structure determination

All diffraction data were collected on beamline BL17U at the Shanghai Synchrotron Research Facility (SSRF, Shanghai, China) and processed with HKL2000 (Otwinowski and Minor, 1997). All structures were determined by the molecular replacement method with the human ISG15 structure (PDB ID: 1Z2M) and

NS1B-NTR structure (PDB ID: 1XEQ) as search models, respectively, using the program PHASER (McCoy et al., 2007). Subsequent rounds of model building and iterative refinement were carried out with Coot and PHENIX, respectively (Adams et al., 2002; Emsley and Cowtan, 2004).All structural figures were rendered using PyMOL (http://www.pymol.org/).

Isothermal titration calorimetry

Calorimetric experiments were carried out at 25 ℃ using a MicroCal iTC200 instrument (Malvern, UK). All protein samples were exchanged into ITC buffer containing 10mM HEPES-sodium, 500mM NaCl, pH 7.2, by gel filtration. Protein concentration was measured on the basis of its $UV_{280\ nm}$ absorbance. Approximately 0.5 mM ISG15 protein was injected into the stirred calorimeter cell containing 250 μl of 0.05 mM NS1B-NTR in 20 aliquots comprising 2 μl at 2.5-min intervals. All the calorimetric titration data were analyzed using the Origin 7.0 software (OriginLab Corp., USA) according to the "one set of sites" fitting model.

Transfection Assays.

The cDNA encoding full-length NS1B was subcloned into the pcDNA 3.1 vector (Thermo Fisher Scientific, USA) with a C-terminal hemagglutinin (HA) tag. The plasmid expressing ISG15 with an N-terminal 3xFlag tag was constructed by cloning the cDNA encoding ISG15 into the p3xFLAG-CMV-7.1 vector (Sigma-Aldrich, USA). All point mutants in ISG15 cDNA were generated by PCR. The cDNA of

human and mouse E1/E2/E3 conjugating enzymes were cloned into pcDNA 3.1 vector (Thermo Fisher Scientific, USA), yielding constructs of hUbe1L and mUbe1L with a C-terminal Myc tag, constructs of hUbcH8 and mUbcH8 with an N-terminal Myc tag and constructs of hHerc5 and mHerc6 with a C-terminal His tag.

For analysis of NS1B/ISG15 interaction, HEK293T cells were plated on 10-cm dishes and cotransfected with a plasmid expressing 3xFlag-ISG15 and a second plasmid expressing NS1B-HA by using Neofect™ (Neofect, China). The cells were harvested at 36h after transfection and were resuspended in ice-cold lysis buffer (50mM Tris-HCl pH 8.0; 150mM NaCl; 1mM phenylmethylsulfonyl fluoride). After sonication, the cell lysate was cleared by centrifugation at 13000xg for 20 min at 4 ℃. The supernatant was incubated with 10 μL of anti-Flag-tag M2 antibody-immobilized agarose beads (Sigma-Aldrich, USA) for 6 h at 4 ℃. The beads was washed 3 times with 1 mL of lysis buffer and the bound proteins were eluted with SDS sample buffer. The precipitates were subjected to SDS-PAGE followed by western blotting with anti-HA or anti-Flag antibody.

To determine whether the two amino acids in ISG15 at the binding site II of NS1B-NTR/ISG15 complex is critical for the inhibition of ISG15 conjugation, HEK293T cells were grown on 10-cm dishes and transfected with the plasmid constructs of human or mouse E1/E2/E3 conjugating enzymes, NS1B-HA and 3xFlag-ISG15. The cells were treated similarly as described above and the immunoprecipitates were analyzed by immunoblots probed with anti-Flag antibody.

SUPPLEMENTARY FIGURE LEGENDS

Supplementary Fig 1. Structure superimposition of NS1B-NTR and the Ubl domains of different ISG15 proteins.

(A) NS1B-NTR dimer from hISG15/NS1B-NTR (green) (PDB code: 3RT3), mISG15 /NS1B-NTR (yellow), cISG15/NS1B-NTR (magenta), bISG15/NS1B-NTR (cyan) superimposed with unbound NS1B-NTR dimer (red) (PDB code: 1XEQ). (B) Zoomed-in view of the C-terminal tail of NS1B-NTR in complex with hISG15 (green), mISG15 (yellow), cISG15 (magenta), bISG15 (cyan), and in the unbound state (red). (C) Structure superimposition of the N-Ubl domains of hISG15 (green) (PDB code: 3RT3), mISG15 (yellow), cISG15 (magenta) and bISG15 (cyan). (D) Structure superimposition of the C-Ubl domains of hISG15 (green) (PDB code: 3RT3), mISG15 (yellow), cISG15 (magenta) and bISG15 (cyan).

Supplementary Fig 2. The intrinsic flexibility between the two Ubl domains of ISG15 proteins. (A-B) The two cISG15 molecules (A) and bISG15 molecules (B) were overlaid by aligning their respective N-Ubl domain. The flexibility was probed by measuring the angle between the point of divergence and the farthest C terminus. (C) Crystal structures of hISG15 (PDB: 3RT3), mISG15, cISG15 molecule A, cISG15 molecule B, bISG15 molecule A and bISG15 molecule B after alignment based on the N-terminal Ubl domain. Green, hISG15; yellow, mISG15. Magenta, cISG15 molecule A; pink, cISG15 molecule B; cyan, bISG15 molecule A; pale cyan, bISG15 molecule

B.

Supplementary Fig 3. Sequence alignment of ISG15 proteins from the indicated mammalian species. The sequence numbering ruler is based on human ISG15. The secondary structural elements are indicated above the alignment. The conserved leucine in binding site I is boxed in purple. The linker region is boxed in yellow and the first two residues in binding site II are shown in blue. Conserved residues are boxed in red.

Supplementary Fig 4. The binding interfaces in ISG15/NS1B-NTR complexes. (A-D) Overall view of the binding interfaces in ISG15/NS1B-NTR complexes. The NS1B-NTR dimer is shown in surface representation. Binding site I is around the Met9–Gly12 loop in hISG15 (A), Met9–Gly12 loop in mISG15 (B), Met15–Gly18 loop in cISG15 (C) and Met9–Gly12 loop in bISG15 (D). Binding site II is around the first two residues, which are Asp76-Lys77 in hISG15 (A), Gln74-Asn75 in mISG15 (B), Lys82-Asn 83 in cISG15 (C) and Gln76-Asn77 in bISG15 (D) in the linker connecting the N-Ubl and C-Ubl of ISG15 proteins. Red, NS1B-NTR monomer A; blue, NS1B-NTR monomer B; green, hISG15; yellow, mISG15; magenta, cISG15; cyan, bISG15.

Supplementary Fig 5. Site II determines the species preference of NS1B-NTR in binding ISG15 proteins.

Measurement of the binding affinities between NS1B-NTR and hISG15-L10A (A), mISG15-L10A (B), cISG15-L16A (C), bISG15-L10A (D) by ITC.

SUPPLEMENTARY FIGURES

Supplementary Fig 1.

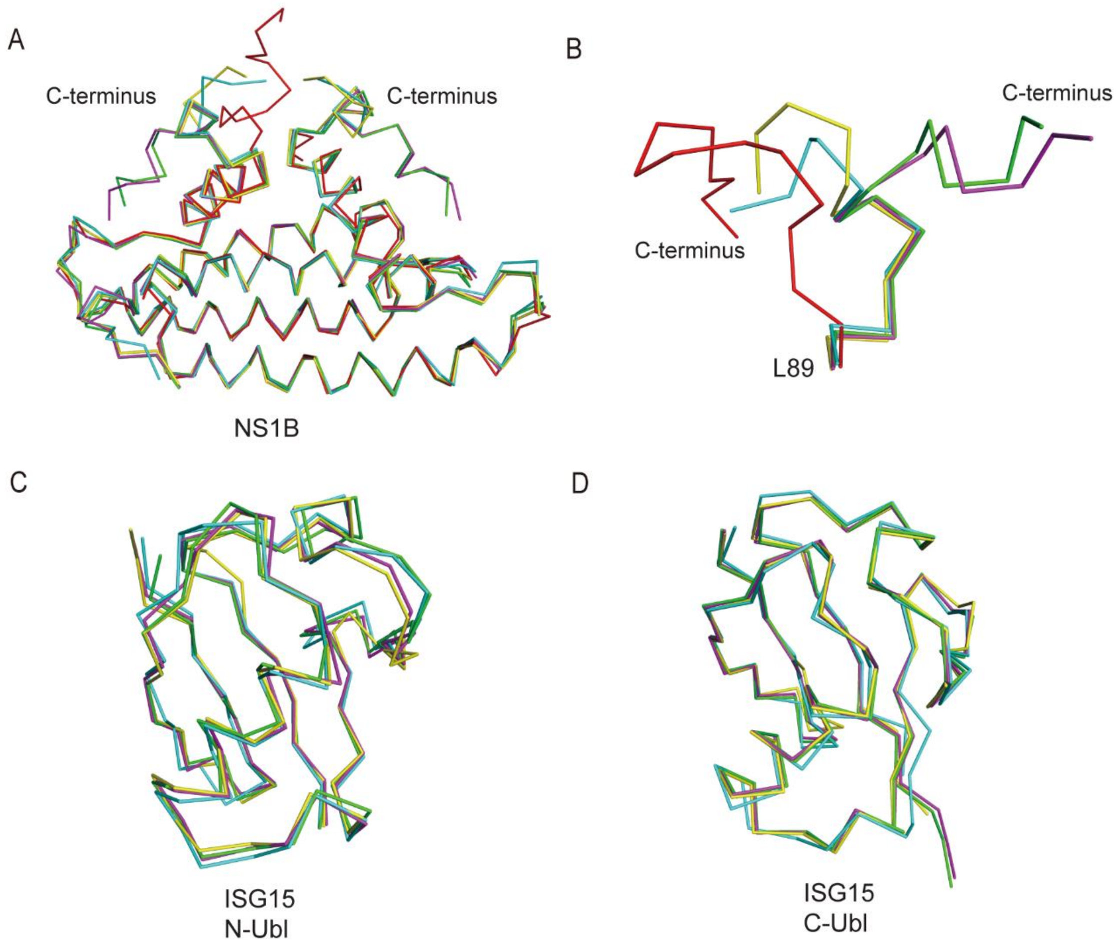

Supplementary Fig 2

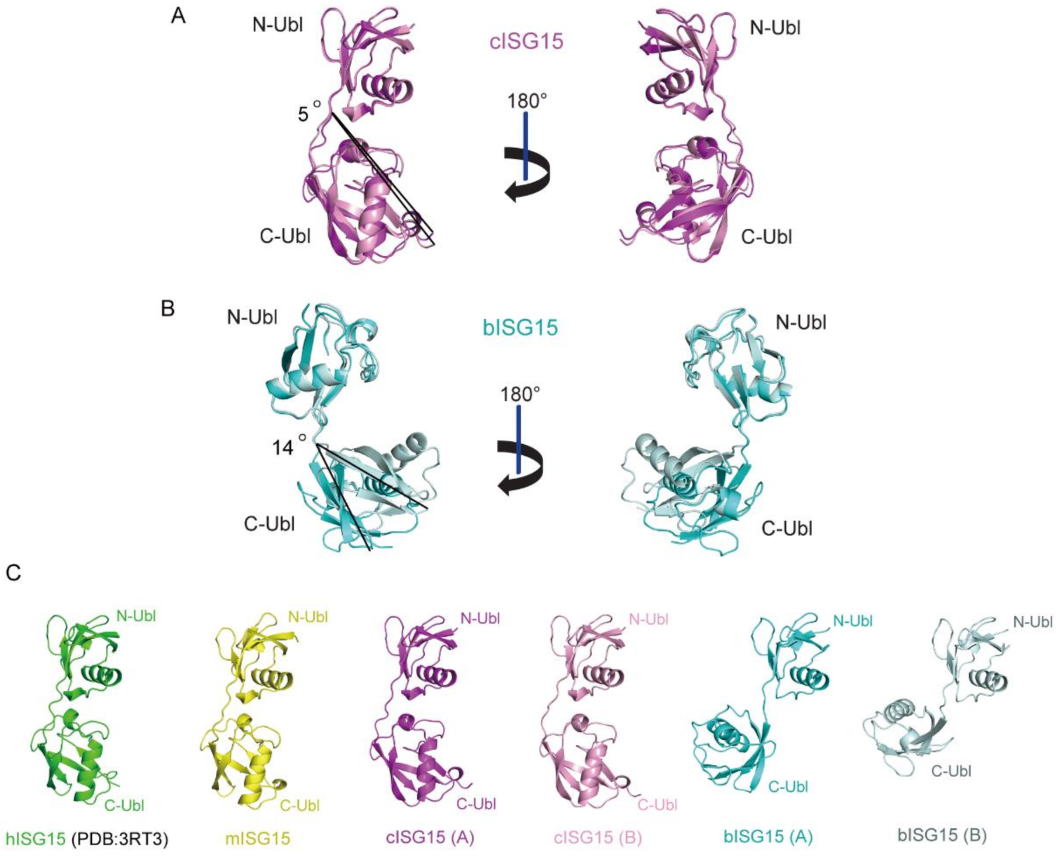

Supplementary Fig 3

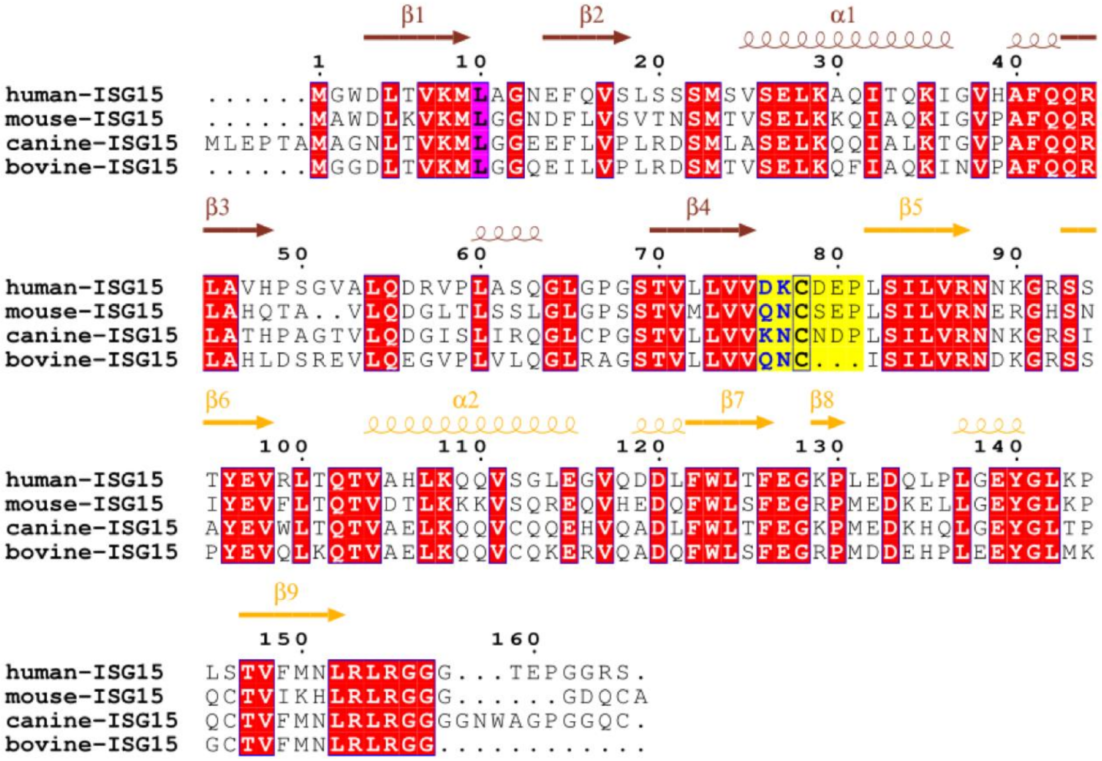

Supplementary Fig 4.

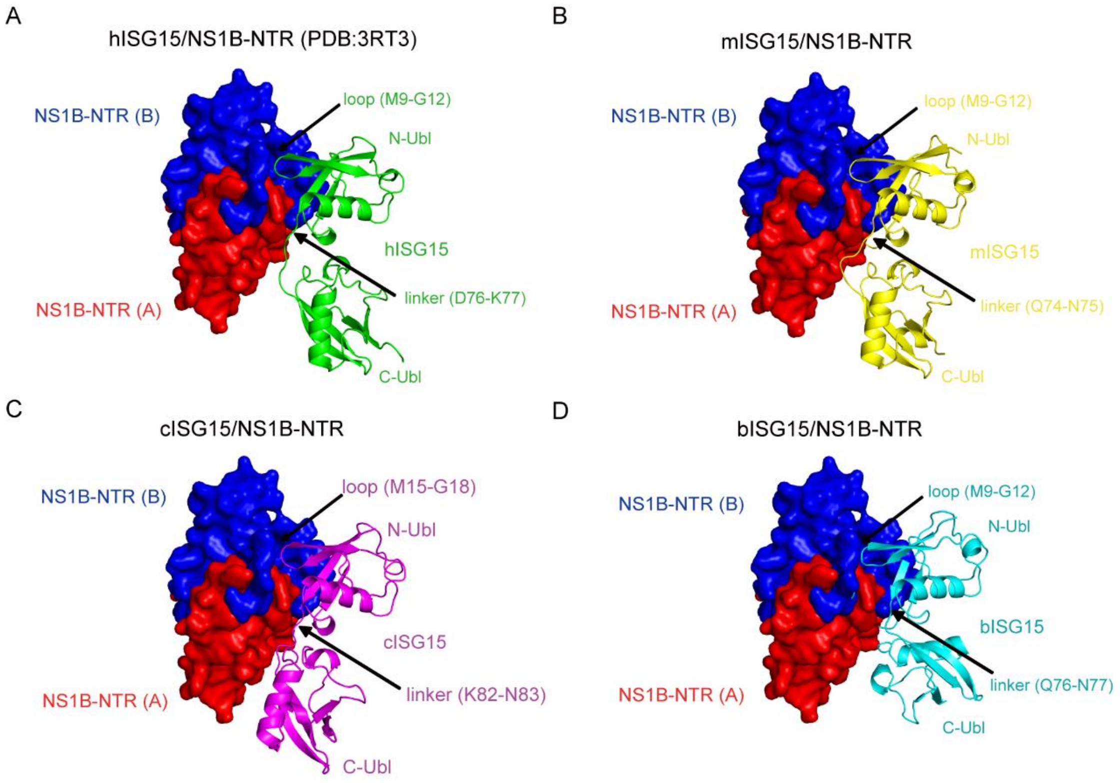

Supplementary Fig 5

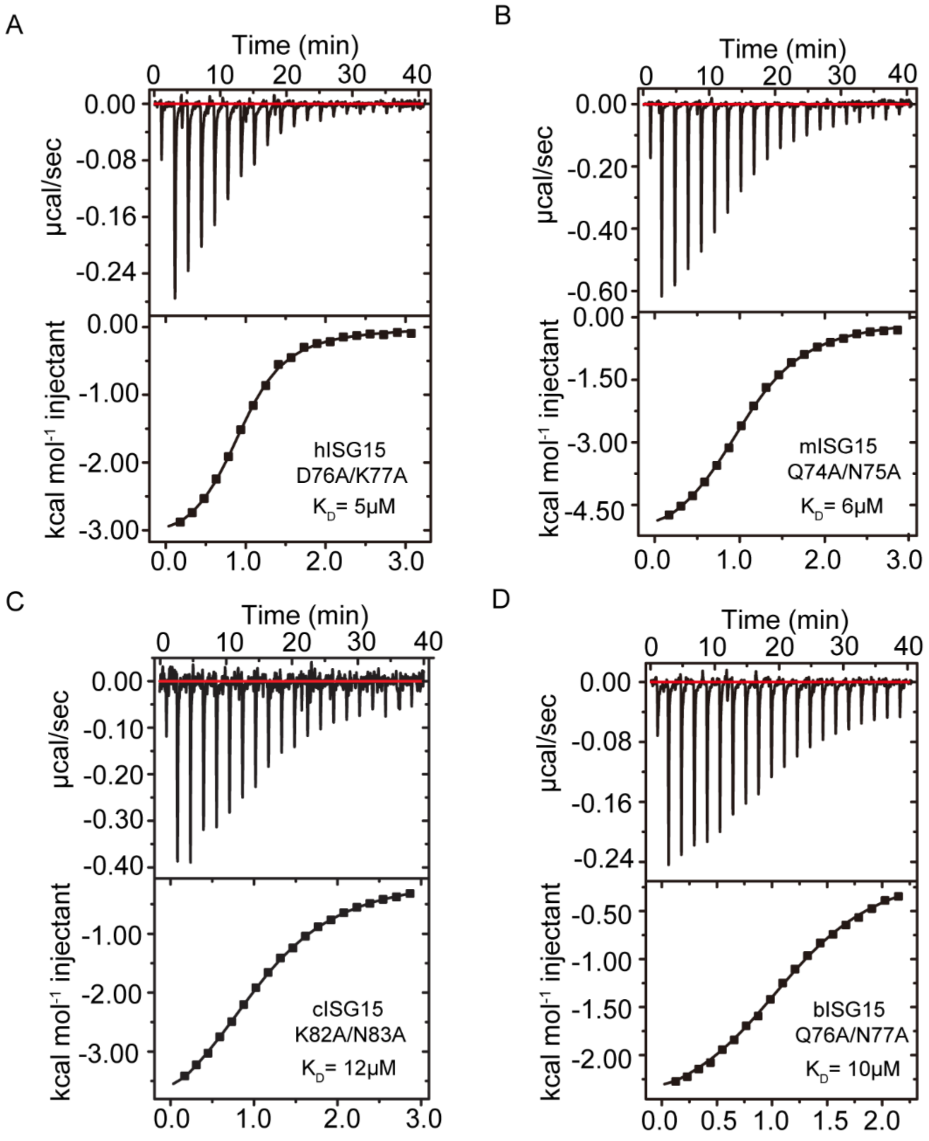

Supplementary Table 1.

Thermodynamic parameters of the interaction of NS1B-NTR with different wild-type and mutant ISG15 proteins.

	N	K	ΔH	ΔS
hISG15	0.957±0.00241 Sites	3.54E6±2.15E5 M^{-1}	-4342±17.41 cal/mol	15.4 cal/mol/deg
mISG15	0.952±0.0199 Sites	7.24E4±3.07E3 M^{-1}	-4117±109.2 cal/mol	8.43 cal/mol/deg
cISG15	1.18±0.0132 Sites	2.88E4±1.25E3 M^{-1}	-3236±67.30 cal/mol	9.55 cal/mol/deg
bISG15	0.819±0.0134 Sites	8.69E4±4.86E3 M^{-1}	-3263±72.48 cal/mol	11.7 cal/mol/deg
hISG15-L10A	0.871±0.0133 Sites	6.03E4±1.60E3 M^{-1}	-9673±187.6 cal/mol	-10.6 cal/mol/deg
hISG15-D76A-K77A	0.933±0.00644 Sites	2.50E5±1.02E4 M^{-1}	-3278±30.78 cal/mol	13.7 cal/mol/deg
mISG15-Q74A-N75A	1.07±0.00640 Sites	1.60E5±4.91E3 M^{-1}	-5637±46.82 cal/mol	4.91 cal/mol/deg
cISG15-K82A-N83A	1.12±0.00604 Sites	8.03E4±1.60E3 M^{-1}	-4607±35.83 cal/mol	6.99 cal/mol/deg
bISG15-Q76A-N77A	1.23±0.0154 Sites	1.03E5±8.32E3 M^{-1}	-2670±53.06 cal/mol	14.0 cal/mol/deg
hISG15-D76Q-K77N	0.992±0.0193 Sites	5.89E4±4.29E3 M^{-1}	-2716±78.91 cal/mol	12.7 cal/mol/deg
mISG15-Q74D-N75K	0.932±0.00268 Sites	4.91E6±3.82E5 M^{-1}	-7605±35.82 cal/mol	5.11 cal/mol/deg
cISG15-K82D-N83K	0.950±0.00301 Sites	3.39E6±2.47E5 M^{-1}	-6856±35.42 cal/mol	6.88 cal/mol/deg
bISG15-Q76D-N77K	0.968±0.0119 Sites	1.26E6±7.06E4 M^{-1}	-7861±43.51 cal/mol	1.55 cal/mol/deg

Supplementary Table 2.

Data collection and refinement statistics

	mISG15/NS1B-NTR	cISG15/NS1B-NTR	bISG15/NS1B-NTR
**Data collection**			
Beamline	SSRF BL17U	SSRF BL17U	NSRRC TPS05A
Wavelength (Å)	0.9796	0.9796	0.9796
Space group	C121	$P4_32_12$	$P2_12_12_1$
Cell dimensions			
a, b, c (Å)	108.11, 37.76, 72.53	77.29, 77.29, 217.58	59.29, 80.17, 109.92
α，β，γ (°)	90.00, 101.34, 90.00	90.00, 90.00, 90.00	90.00, 90.00, 90.00
Resolution (Å)	35.58-2.48 (2.57-2.48)	34.57-2.40 (2.48-2.40)	33.33-3.00 (3.10-3.00)
R_{merge} (%)	12.0 (87.1)	13.8 (243.2)	8.2 (91.2)
I/σI	14.4 (5.1)	20.7 (1.8)	15.9 (1.7)
Completeness (%)	98.0	100.0	98.0
Redundancy	8.3 (8.4)	11.5 (11.9)	5.2 (1.5)
**Refinement**			
Resolution (Å)	35.58-2.48 (2.57-2.48)	34.57-2.40 (2.48-2.40)	33.33-3.00 (3.10-3.00)
No. reflections	10221 (907)	26523 (2615)	10761 (909)
R_{work}/R_{free} (%)	20.4/27.7 (30.54/35.73)	20.72/26.56 (27.68/30.40)	23.15/24.29 (33.30/29.12)
No. of atoms			
Protein	1925	3954	3796
Average B factor ($Å^2$)	94.27	71.01	87.01
r.m.s. deviations			
Bond lengths (Å)	0.008	0.008	0.011
Bond angles (Å)	0.99	1.05	1.45
Ramachandran plot (%)			
Favored	93.0	98.0	89.0
Allowed	6.3	1.8	7.4
Disallowed	0.42	0.20	3.2

Supplementary Table 3.

Interactions (d ≤ 4.0 Å) at the mISG15/NS1B-NTR binding interface.

NS1B-NTR / mISG15	Monomer A	Monomer B
M9	Q37	
L10	A39	M84, V87, L88
G11		A19, M84
I36	R38	
G37	R38	
V38	R38	
R44		M91, D92, P93
A46		P93
T49		I97
M70		P93
V72	Q37	V87, P93
V73	W36, Q37	M91
Q74	W36	M91
N75	W36	
E78	E75	

Supplementary Table 4.

Interactions (d ≤ 4.0 Å) at the cISG15/NS1B-NTR binding interface.

NS1B-NTR / cISG5 monomer A	Monomer A	Monomer B	NS1B-NTR / cISG5 monomer A	Monomer A	Monomer B
K14		F100, E101	K14		F100, E101
M15	Q37	F100	M15	Q37	F100
L16	A39	M84, V87, L88, F100	L16	A39	M84, V87, L88, F100
G17		A19, M84, G99, E101	G17		A19, M84, G99, E101
G18		F100	G18		F100, E101, P102
V44	R38		V44	R38	
P55		I97	A52		P93
A56		S94	P55		I97
L78		P93, F100	A56		S94
L79		F100	L78		P93, F100
V80	Q37	M91, F100	L79		F100
V81	W36, Q37	M91	V80	Q37	M91, P93, F100
K82		M91	V81	W36, Q37	M91
N83	W36		K82		M91
N85	E32, W36		N83	W36	

Supplementary Table 5.

Interactions (d ≤ 4.0 Å) at the bISG15/NS1B-NTR binding interface.

NS1B-NTR / bISG15 monomer A	Monomer A	Monomer B	NS1B-NTR / bISG15 monomer A	Monomer A	Monomer B
M9	Q37		M9	Q37	
L10	A39	A19, M84, V87, L88	L10		A19, M84, V87, L88
G11	A39	P14, G15, A19	G11	A39	P14, G15, A16, A19
Q13	Q37, R38		Q13	Q37, R38	
I36	R38		I36	R38	
N37	R38		V38	R38	
R44		F90, M91, D92	R44		F90, M91, D92
A46		P93	A46		P93
L48		P93, F100	S50		E98
D49		I97	R51		S94, I97
R51		S94, I97, E98	L72		P93
V53		D92	V74	Q37	M91, P93
L72		P93	V75	Q37	
V74	Q37	M91, P93	Q76		M91
V75	W36, Q37		N77	W36	
Q76		M91	Q96		M91
N77	W36		K98	E29	
K98	E29, R33		A102	E98	
H132	E76		E103	E98	
E135	E75, K78				
E136	S73, E76				

REFERENCE

Adams, P.D., Grosse-Kunstleve, R.W., Hung, L.W., Ioerger, T.R., McCoy, A.J., Moriarty, N.W., Read, R.J., Sacchettini, J.C., Sauter, N.K., and Terwilliger, T.C. (2002). PHENIX: building new software for automated crystallographic structure determination. Acta Crystallogr D Biol Crystallogr 58, 1948-1954.

Emsley, P., and Cowtan, K. (2004). Coot: model-building tools for molecular graphics. Acta Crystallogr D Biol Crystallogr 60, 2126-2132.

McCoy, A.J., Grosse-Kunstleve, R.W., Adams, P.D., Winn, M.D., Storoni, L.C., and Read, R.J. (2007). Phaser crystallographic software. J Appl Crystallogr 40, 658-674.

Otwinowski, Z., and Minor, W. (1997). Processing of X-ray diffraction data collected in oscillation mode. Methods Enzymol 276, 307-326.
